# Adaptive Virtual Impedance Droop Control of Parallel Inverters for Islanded Microgrids

**DOI:** 10.3390/s25165166

**Published:** 2025-08-20

**Authors:** Hongzhi Yang, Zibo Sun, Haoran Wang, Yipei Wang, Mengmei Zhu, Lei Guo, Guangxu Zhou, Hongzhang Lyu

**Affiliations:** 1 Institute of Automation, Qilu University of Technology (Shandong Academy of Sciences), Jinan 250000, China; sd_hongzhi_yang@163.com (H.Y.); 10431230602@stu.qlu.edu.cn (Z.S.); wanghaoran131@sina.com (H.W.); wangyipei@qlu.edu.cn (Y.W.); zmm2021@163.com (M.Z.); guoleihaishi@sina.cn (L.G.); 2 Qingdao Veccon Electric Co., Ltd., Qingdao 266200, China; hongzhang.lyu@veccon.com.cn

**Keywords:** adaptive virtual impedance, droop control, parallel inverters, power sharing

## Abstract

The droop control strategy, known for its communication-free nature, is widely adopted for the parallel operation of inverter units. However, in microgrids, mismatches in line impedances and various measurement inaccuracies often lead to imbalanced reactive power sharing among inverters and significant circulating current. To address these challenges, this paper proposes an adaptive droop control method that relies solely on local measurements from each inverter, eliminating the need for communication. The proposed approach integrates the deviation between the ratio of reactive power to output voltage and its reference value to generate an Adaptive Virtual Impedance Droop Control (AVIDC) mechanism. This enables a dynamic balance between reactive power output and voltage drop. The simulation and experimental results validate the effectiveness of the proposed control strategy, demonstrating a significant improvement in the accuracy of reactive power sharing.

## 1. Introduction

**Figure 1:**
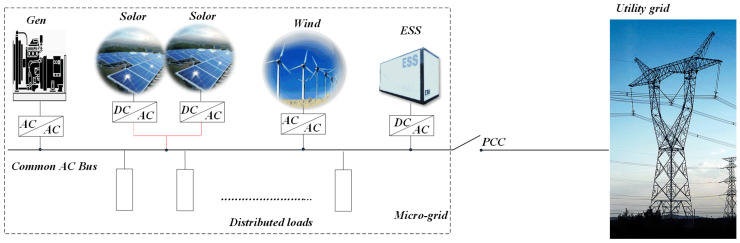
Microgrid structure.

Microgrids are localized low-voltage power supply systems that coexist with the conventional power grid. Their design focuses on generating electricity at the point of consumption, aiming to minimize transmission losses and maximize the efficiency of the generated power. Power electronic inverters facilitate the smooth integration and dependable functioning of distributed energy resources (DERs), thereby presenting substantial prospects for extensive implementation [1,2,3]. Three-level inverters, a type of multilevel inverter, possess advantages such as reduced Total Harmonic Distortion (THD) over traditional two-level inverters, thus finding suitability in a wide range of industrial applications [4]. Figure 1 shows the microgrid architecture, where traditional generators, PV arrays, and battery systems are all connected to a shared AC bus. When the point of common coupling (PCC) is closed, the microgrid remains linked to the main grid; opening the PCC isolates the network, placing it in an islanded operation. Compared to a single distributed generation (DG) unit, microgrids offer greater capacity and enhanced control flexibility, allowing them to meet stringent power-quality requirements [5,6].

In islanded mode, effectively managing the rated power and suppressing circulating currents among DGs is critical. Droop control has become the preferred method owing to its scalability, modular design, built-in redundancy, and operational flexibility. This enables decentralized control and wireless parallel operation of multiple inverters, which is particularly advantageous for DG systems. However, this control strategy also presents several challenges: (1) low accuracy in reactive power sharing, (2) significant circulating currents among parallel units, and (3) voltage and frequency droop at the output of inverter units [7,8,9].

To address the issue of unequal power distribution and enhance the control performance of microgrids, various power-sharing control strategies have been proposed, as discussed in [10], including centralized control, master-slave control, and droop control based on voltage power droop and frequency reactive boost. However, these approaches typically rely on accurate line-impedance information to suppress power coupling, which limits their practical feasibility. To mitigate the impact of line impedance mismatches and improve the power-sharing accuracy, researchers have developed several enhanced droop control strategies. In [11], we reviewed a synchronized fixed-frequency droop control strategy based on a global satellite navigation system, and for the first time, we combined the satellite timing signal with droop control to realize the synchronized operation of a distributed power supply in microgrids without communication. In [12], improvements were made by introducing error reduction and voltage restoration operations to enhance reactive power sharing precision. In [13], the authors devised a self-adaptive control scheme that modifies system inertia and damping in real time to bolster frequency regulation and maintain voltage stability. In [14], a secondary frequency and voltage droop control method based on consensus theory and the concept of Virtual Synchronous Generators (VSGs) was introduced, enhancing system damping and stability. However, its application is limited to ideal conditions and depends heavily on communication links between distributed units. Gabl O. M. A. devised a multi-objective mixed integer nonlinear programming (MINLP) framework that incorporates secondary control zone constraints. Although the approach delivers good performance, it is highly model-dependent and therefore lacks the flexibility needed to cope with complex or rapidly changing operating conditions [15]. In [16], conventional droop control was complemented with voltage- and current-averaging regulators, which curtailed bus voltage deviations and improved the accuracy of load current sharing. Verma V proposed inserting an active power offset to decouple the power components, a step that subsequently refines reactive power allocation [17]. However, this method suffers from a slow dynamic response and high sensitivity to load types.

An alternative approach focuses on using adaptive virtual impedance to directly compensate for mismatches in the line impedance. This method not only improves power sharing performance and enhances the system’s ability to handle nonlinear loads, but also increases oscillation damping and strengthens overall system stability [18,19,20]. Over the past few years, numerous control schemes leveraging adaptive virtual impedance have been introduced. M. Ahmed proposed an adaptive virtual-impedance scheme for voltage-source converters in hybrid AC/DC microgrids; however, the approach overlooked the nonlinear characteristics intrinsic to microgrid environments [21]. In [22], the virtual resistance and reactance are designed as linear functions of the output current of the DG, which improves the conventional droop and inverse droop control, ensuring equal sharing of active and reactive power. R. An proposed a successive-approximation approach to virtual-impedance adjustment that alternates between conventional droop control and adaptive tuning; however, abrupt load changes may cause deviations in the equivalent line impedance [23]. In [24], frequency regulation and inter-inverter power balancing can be attained with a virtual synchronous generator that adjusts its inertia in real time; nevertheless, the method hinges on very precise impedance measurements and comprehensive frequency response characterization. The Distributed Adaptive Virtual Impedance Control (DAVIC) strategy proposed in [25] adjusts virtual impedance automatically based on the difference between local power and the average power across all modules, yet it depends on low-bandwidth CAN communication for data sharing, which introduces time delays. Adib proposed an adaptive inductance-feedforward scheme that preserves system stability even when the grid is weak or exhibits high impedance [26]. In [27], a dynamic model of the PCC was developed to elucidate the power coupling mechanism, and by adaptively tuning droop gains and feedforward coefficients in real time, the dynamic performance of the microgrid was significantly improved.

Existing methods that aim to improve the accuracy of reactive power sharing and minimize circulating currents among distributed units face limitations in terms of real-time adaptability and general applicability, making them inadequate for handling complex and variable line-impedance conditions. This study proposes an adaptive virtual impedance control scheme aimed at equalizing power sharing and eliminating circulating currents in parallel inverters. The principal contributions of this study are summarized as follows:

- By examining the reactive power mismatch caused by conventional droop control in parallel inverter systems, this study paves the way for faster and more accurate reactive power balancing.
- An adaptive virtual impedance control method is introduced, in which an adaptive controller is derived by integrating the deviation between the ratio of reactive power to output voltage and its desired reference value for each measurement unit.
- To validate the effectiveness of the proposed control strategy, a prototype system was designed and constructed. Experimental results demonstrate that the output waveforms of the parallel inverters exhibit strong consistency and that circulating currents between inverter units are effectively suppressed.

The remainder of this paper is organized as follows. Section 2 reviews droop control fundamentals and pinpoints the reactive power sharing errors typical of conventional implementations. Section 3 elaborates on the proposed solution, the Adaptive Virtual Impedance Droop Control (AVIDC), and explains its operating principles. Section 4 presents the simulation and laboratory evidence that verifies the effectiveness of the AVIDC. Finally, Section 5 concludes the paper with a summary of the key findings.

## 2. Conventional Droop Control and Analysis of Power Sharing Errors

**Figure 2:**
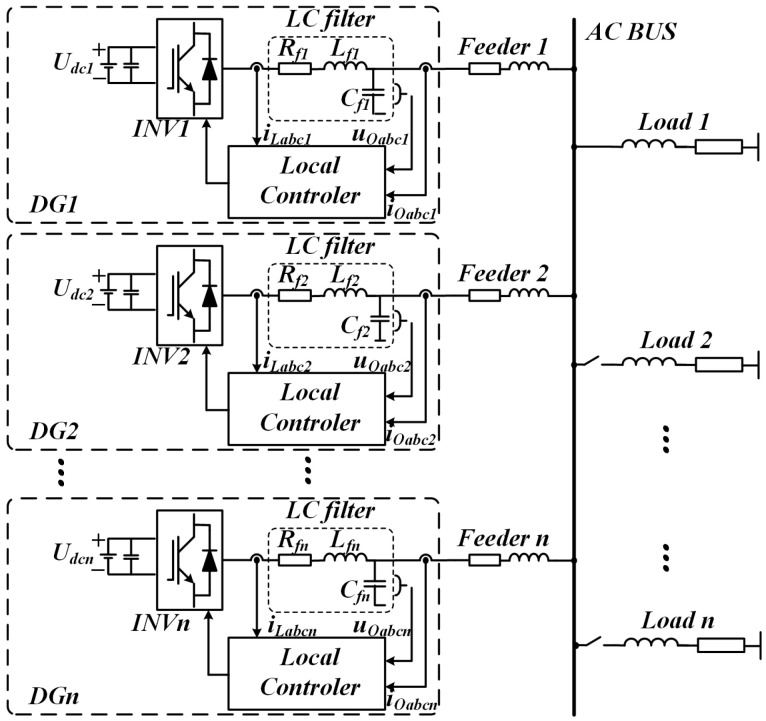
Topology of an isolated microgrid containing n DG units.

The parallel operation structure of multiple DG units is illustrated in Figure 2. In islanded mode, each DG unit is equipped with an independent controller. Considering a single DG unit as an example, the main circuit employs a simplified DC voltage source *U_dcn_* to represent the distributed energy source. The inverter *INV_n_* converts the DC voltage into three-phase AC voltage and current. The output stage includes a filter composed of inductance *L_fn_* and capacitance *C_fn_*, with *R_fn_* representing the equivalent series impedance. Each DG unit is connected to the common AC bus via transmission lines *Feeder_n_* (*n* = 1,2,3,…), forming a parallel network with the other DG units to complete the microgrid topology [28].

### 2.1. Conventional Droop Control Method

In islanded mode, droop control enables each DG unit to operate independently, thereby realizing a true “plug-and-play” capability without the need for internal communication. This study focuses on a droop control scheme that links active power to frequency (*P*–*ω*) and reactive power to voltage (*Q*–*U*), assuming that the output impedance of the inverter is mainly inductive.

**Figure 3:**
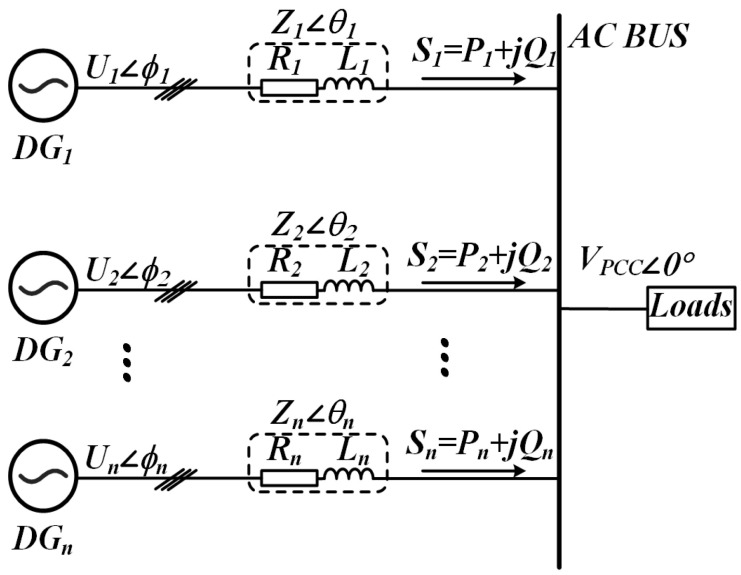
Conceptual schematic of an isolated microgrid configuration.

A simplified circuit of an islanded microgrid consisting of n DGs is shown in Figure 3. The complex power output *S_n_* of a single DG unit can be expressed as:

(1) $$S_{n}=P_{n}+jQ_{n}$$

where *P_n_* and *Q_n_* represent the active and reactive powers, respectively, and are given by the following expressions [29]:

(2) $$P_{n}=\frac{U_{n}V_{PCC}}{Z_{n}}\cos(\theta_{n}-\phi_{n})-\frac{V_{PCC}^{2}}{Z_{n}}\cos\theta_{n}$$

(3) $$Q_{n}=\frac{U_{n}V_{PCC}}{Z_{n}}\sin(\theta_{n}-\phi_{n})-\frac{V_{PCC}^{2}}{Z_{n}}\sin\theta_{n}$$

where *U_n_* signifies the inverter output voltage amplitude, and *ϕ_n_* specifies its power angle. Variable *V_pcc_* denotes the voltage magnitude measured at PCC. The output impedance is characterized by *Z_n_* as its magnitude and *θ_n_* as its phase.

Given that sizable filter inductors are usually employed, the inverter output impedance can be regarded as chiefly inductive—the impedance *Z_n_* = *R_n_* + *jX_n_* can be approximated as *Z_n_* ≈ *jX_n_*. Under this assumption, Equations (2) and (3) can be simplified as follows [30]:

(4) $$P_{n}=\frac{U_{n}V_{PCC}}{X_{n}}\phi_{n}$$

(5) $$Q_{n}=\frac{U_{n}V_{PCC}-V_{PCC}^{2}}{X_{n}}$$

where *X_n_* represents the output reactance of the inverter.

**Figure 4:**
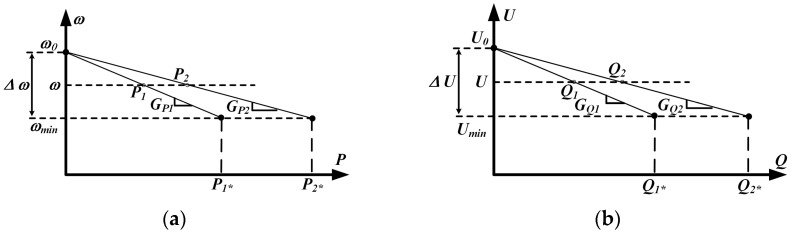
(**a**) *P*–*ω* droops; (**b**) *Q*–*U* droops.

Drawing on these expressions and recognizing that the phase offset between *U_n_* and *V_pcc_* is negligible (sin*ϕ* ≈ *ϕ* and cos*ϕ* ≈ 1), it can be seen that active power *P_n_* is acutely responsive to variations in the power angle, whereas reactive power *Q_n_* is governed chiefly by differences in voltage magnitude. As a result, most communication-free load-sharing controllers incorporate a voltage droop *U* into the voltage reference of the inverter. To replicate this behavior, droop control schemes labeled (*P*–*ω*) and (*Q*–*U*), as shown in Figure 4, are typically employed. Accordingly, the inverter output voltage reference, incorporating both its angular frequency and amplitude, can be expressed as follows [31]:

(6) $$\omega_{n}=\omega_{0}-G_{Pn}P_{n}$$

(7) $$U_{n}=U_{0}-G_{Qn}Q_{n}$$

where *ω*_0_ and *U*_0_ denote the inverter’s nominal angular frequency and output voltage magnitude, respectively, and *G_pn_* and *G_Qn_* serve as the droop gains for active and reactive power. The corresponding frequency and voltage droop coefficients are obtained using the following expressions:

(8) $$G_{Pn}=\frac{\Delta \omega }{{P_{n}}^{*}}$$

(9) $$G_{Qn}=\frac{\Delta U}{{Q_{n}}^{*}}$$

where Δ*ω* and Δ*U* represent the maximum allowable deviations in frequency and voltage, respectively. P_n_^*^ and Q_n_^*^ denote the nominal active and reactive power values at that operating point.

### 2.2. Reactive Power Analysis of Conventional Droop Control

Rearranging Equation (5) yields an alternative formulation for the output voltage magnitude of the distributed generator, as follows:

(10) $$U_{n}=V_{\mathrm{PCC}}+\frac{X_{n}Q_{n}}{V_{\mathrm{PCC}}}$$

**Figure 5:**
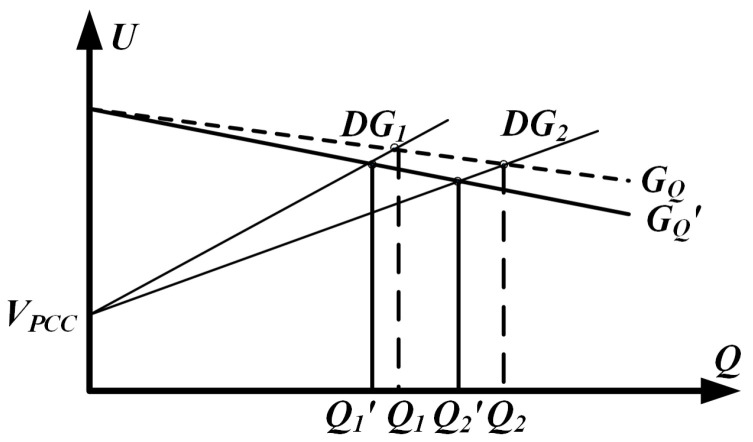
Parallel DGS output Q-U loop and control.

Figure 5 illustrates the *Q*–*U* curve associated with droop control, which is employed to investigate how reactive power is shared among sources. In practical deployments, the different geographical positions of the DG units lead to unequal line impedances. To simplify the analysis, a two-DG system is first considered and then generalized to an n-DG configuration. According to Equation (10), when line impedance mismatches occur, the power characteristic curves of DG1 and DG2 exhibit different slopes, leading to errors in reactive power sharing. Although steepening or flattening the droop curve can reduce current-sharing errors and yield a more precise reactive power allocation, the adjustment introduces a pronounced voltage droop that ultimately compromises overall system stability.

Proportional reactive power allocation across DG units is attained by choosing each unit’s *Q*–*U* droop coefficient to vary inversely with its rated reactive power capability. That is:

(11) $$n_{1}Q_{1}^{*}=n_{2}Q_{2}^{*}$$

The voltage difference between two distributed generators leads to reactive power sharing errors and circulating currents. The relative reactive power error rate *e_Qn_* can be derived as follows:

(12) $$e_{Qn}=\frac{\Delta Q_{n}}{Q_{n}^{*}}=\frac{Q_{n}-Q_{n}^{*}}{Q_{n}^{*}}\times 100\%$$

By combining Equations (7) and (10), the reactive power delivered by a DG unit can be expressed as:

(13) $$Q_{n}=\frac{V_{\mathrm{PCC}}(U_{0}-V_{\mathrm{PCC}})}{\left(X_{n}+G_{Q}V_{\mathrm{PCC}}\right)}$$

From Equation (13), we can derive the following equation:

(14) $$(Q_{1}^{*}+\Delta Q_{1})(X_{1}+G_{Q1}V_{\mathrm{PCC}})=(Q_{2}^{*}+\Delta Q_{2})\left(X_{2}+G_{Q2}V_{\mathrm{PCC}}\right)$$

Under steady state conditions, a pair of parallel DG units must deliver a fixed total amount of reactive power. Generalizing to any number of DG units, the algebraic sum of their reactive power sharing errors must therefore equal zero, as indicated by

(15) $$\Delta Q_{1}+\Delta Q_{2}=0$$

Based on Equations (10)–(15), the reactive power sharing error *e_Qn_* for two DG units operating in parallel is expressed as

(16) $$e_{Q1}=\frac{G_{Q1}X_{2}-G_{Q2}X_{1}}{G_{Q2}\left[X_{1}+X_{2}+G_{Q1}V_{\mathrm{PCC}}+G_{Q2}V_{\mathrm{PCC}}\right]}$$

(17) $$e_{Q2}=\frac{G_{Q2}X_{1}-G_{Q1}X_{2}}{G_{Q1}\left[X_{1}+X_{2}+G_{Q1}V_{\mathrm{PCC}}+G_{Q2}V_{\mathrm{PCC}}\right]}$$

For two DG units with different capacities, a sufficient condition for achieving balanced reactive power sharing is

(18) $$\begin{array}{c} \frac{Q_{1}^{*}}{Q_{2}^{*}}=\frac{X_{2}}{X_{1}} \end{array}$$

Although line impedance is difficult to measure accurately in practical microgrid operations, a desirable performance can still be achieved through the adoption of appropriate control strategies.

## 3. Proposed AVIDC Method

### 3.1. Design of Equivalent Line Impedance

The output impedance of the closed-loop inverter is pivotal for accurate power sharing and underpins an effective droop control design. By judiciously shaping this impedance, one can counteract line impedance mismatches that would otherwise degrade performance. The total output impedance comprises three parts: the intrinsic impedance of the inverter, added virtual impedance, and feeder impedance. Of these, the intrinsic component dominates and exerts the greatest influence on the system stability and dynamic behavior.

Based on the model of the inverter’s main circuit, it is evident that coupling exists between the *d*-axis and *q*-axis components after coordinate transformation. To eliminate this coupling and achieve decoupled control of the *dq*-axis components, feedforward decoupling is introduced during the design of the voltage and current control loops. The outer voltage loop employs a proportional-integral controller *G_V_* = *K_pv_* + *K_iv_*/*s*, which ensures a stable voltage output. The inner current loop utilizes a proportional controller *G_i_* = *k_pc_*, which enhances the dynamic response of the system [22].

**Figure 6:**
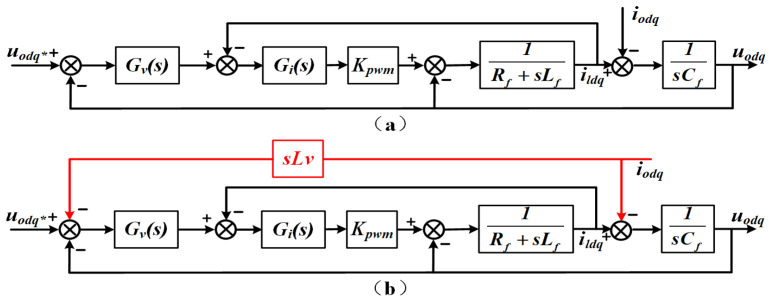
Transfer function schematic of the inverter (**a**) without virtual inductance (**b**) incorporating virtual inductance.

**Figure 7:**
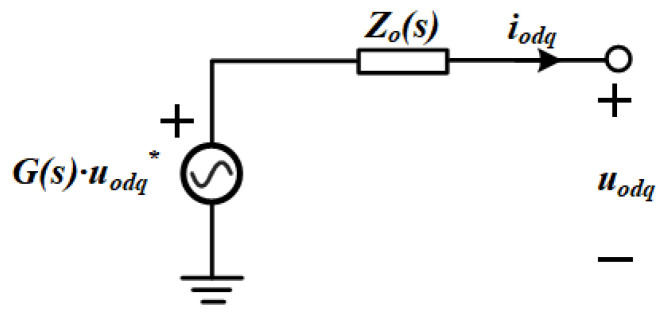
Thévenin equivalent representation of the closed-loop configuration.

Figure 6a presents a simplified equivalent control diagram for the decoupled voltage-current dual loop system. The corresponding closed-loop transfer function is therefore expressed as

(19) $$u_{odq}=G_{u}(s)\cdot u_{odq}^{*}-Z_{o}(s)\cdot i_{odq}$$

where *G_u_*(*s*) represents the voltage gain and *Z_o_*(*s*) denotes the output impedance, defined as follows:

(20) $$G_{u}(s)=\frac{K_{p\nu }G_{i}K_{pwm}s+K_{i\nu }G_{i}K_{pwm}}{C_{f}L_{f}s^{3}+C_{f}(R_{f}+G_{i}K_{pwm})s^{2}+(K_{p\nu }G_{i}K_{pwm}+1)s+K_{i\nu }G_{i}K_{pwm}}$$

(21) $$Z_{o}(s)=\frac{L_{f}s^{2}+(R_{f}+G_{i}K_{pwm})s}{C_{f}L_{f}s^{3}+C_{f}(R_{f}+G_{i}K_{pwm})s^{2}+(K_{pv}G_{i}K_{pwm}+1)s+K_{iv}G_{i}K_{pwm}}$$

where *K_pv_* represents the voltage loop proportional gain, *K_iv_* the voltage loop integral gain, *G_i_* the current loop proportional gain, and *K_pwm_* an inverter gain factor that scales with the output voltage. With these parameters defined, the inverter can be represented as a two-terminal Thévenin equivalent circuit, as depicted in Figure 7.

The behavior of *G_u_*(*s*) is directly related to the voltage stability. Under ideal tuning, the controller gains are set such that *G_u_*(*s*) = 1 and *Z_o_*(*s*) = 0, allowing the output voltage to follow its reference exactly. However, in real low-voltage microgrids, feeder impedances comprise both resistive and inductive elements, making it challenging to separate active and reactive power regulation.

To ensure that the total output impedance remains predominantly inductive, a virtual inductance *L_v_* is introduced into the control block diagram and tuned around its nominal value, as shown in Figure 6b. The product of the inverter output current and virtual inductance is used as a negative feedback signal applied to the voltage reference. According to Thévenin’s theorem, Equation (19) can be rewritten as

(22) $$\begin{array}{ll} u_{odq}^{\prime } & =G_{u}\left(s\right)\cdot u_{odq}^{*}-Z_{o}^{\prime }(s)i_{odq}\\ & =G_{u}\left(s\right)\cdot u_{odq}^{*}-\left(Z_{o}\left(s\right)+G_{u}\left(s\right)\cdot sL_{v}\right)i_{odq} \end{array}$$

**Figure 8:**
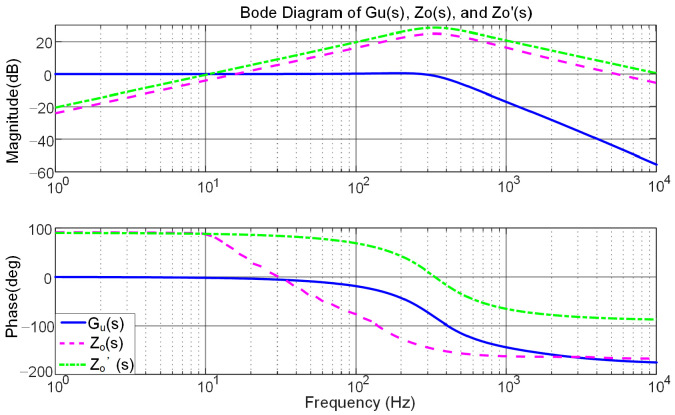
Bode plots showing the voltage gain response of the system and its corresponding equivalent impedance.

System and controller parameters are listed in Table 1. Figure 8 displays the magnitude and phase response curves of variables *G_u_*(*s*) and *Z^’^_o_*(*s*), confirming that the closed loop is stable. At the fundamental frequency, the voltage loop gain *G_u_*(*s*) = 1, enabling the output voltage to match its reference without a steady-state error. Moreover, *Z’o*(*s*) has a larger amplitude margin than *Zo*(*s*), resulting in better voltage stabilization when the load changes abruptly, and the high output impedance can suppress the loop current, making the power distribution more accurate. *Z’o*(*s*) has a large phase angle margin, which is less prone to oscillation and overshooting, and the system stability is high. At the same frequency, variable *Z^’^_o_*(*s*) shows a phase lead of roughly 90°, giving the network a much stronger inductive character than in the case of *Z_o_*(*s*). The inclusion of virtual impedance thus achieves effective power decoupling and allows the overall output impedance to be treated as an ideal inductance.

### 3.2. Adaptive Virtual Impedance Droop Control

Since DG units are unable to determine the relative magnitude of their respective line impedances, it is challenging to appropriately adjust the virtual impedance values. To address this issue, an AVIDC method is proposed to compensate for line impedance mismatches. In this approach, the output reactive power and voltage magnitude are fed into an adaptive virtual impedance controller. Virtual impedance tuning is achieved solely through locally measured information without requiring any communication between the DG units.

Because the impedance angle of Δ*L_vir_* can be tuned to account for variations in the feeder impedance, the adaptive virtual impedance is intentionally chosen to be inductive so that the control concept can be more clearly demonstrated. Consequently, the adaptive virtual impedance for each DGn unit (n = 1, 2,…, n) is defined as

(23) $$\Delta L_{\nu ir}=\frac{k_{Q}}{s^{2}}(\frac{Q}{u}-\frac{Q^{*}}{u^{*}})$$

where *k_Q_* is the integral gain used to adjust the virtual inductance, and placing the *Q/u* ratio in the minuend position directly reflects the extent to which the system deviates from its ideal state. This deviation metric guides the system toward the desired operating points. Additionally, second-order integration continuously integrates the error caused by external disturbances, gradually compensating for their effects and enhancing system robustness.

By combining Equations (16) and (23), we can derive the expression for the relative reactive power error e^*^_Q1_ of DG1 after the introduction of virtual impedance:

(24) $$\begin{array}{ll} {e^{*}}_{Q1} & =\frac{G_{Q1}X_{2}-n_{2}X_{1}+\omega_{0}\frac{k_{Q}}{s^{2}}\left(G_{Q1}\left(\frac{Q_{2}}{U_{2}}-\frac{Q_{2}^{*}}{U_{2}^{*}}\right)-G_{Q2}\left(\frac{Q_{1}}{U_{1}}-\frac{Q_{1}^{*}}{U_{1}^{*}}\right)\right)}{G_{Q2}\left[X_{1}+X_{2}+G_{Q1}V_{PCC}+G_{Q2}V_{PCC}+\omega_{0}\frac{k_{Q}}{s^{2}}\left(\frac{Q_{1}}{U_{1}}-\frac{Q_{1}^{*}}{U_{1}^{*}}+\frac{Q_{2}}{U_{2}}-\frac{Q_{2}^{*}}{U_{2}^{*}}\right)\right]}\\ & =e_{Q11}+e_{Q12}\\ e_{Q11} & =\frac{G_{Q1}X_{2}-G_{Q2}X_{1}}{G_{Q2}\left[X_{1}+X_{2}+G_{Q1}V_{PCC}+G_{Q2}V_{PCC}+\omega_{0}\frac{k_{Q}}{s^{2}}\left(\frac{Q_{1}}{U_{1}}-\frac{Q_{1}^{*}}{U_{1}^{*}}+\frac{Q_{2}}{U_{2}}-\frac{Q_{2}^{*}}{U_{2}^{*}}\right)\right]}\\ e_{Q12} & =\frac{\omega_{0}\frac{k_{Q}}{s^{2}}\left(G_{Q1}\left(\frac{Q_{2}}{U_{2}}-\frac{Q_{2}^{*}}{U_{2}^{*}}\right)-G_{Q2}\left(\frac{Q_{1}}{U_{1}}-\frac{Q_{1}^{*}}{U_{1}^{*}}\right)\right)}{G_{Q2}\left[X_{1}+X_{2}+G_{Q1}V_{PCC}+G_{Q2}V_{PCC}+\omega_{0}\frac{k_{Q}}{s^{2}}\left(\frac{Q_{1}}{U_{1}}-\frac{Q_{1}^{*}}{U_{1}^{*}}+\frac{Q_{2}}{U_{2}}-\frac{Q_{2}^{*}}{U_{2}^{*}}\right)\right]} \end{array}$$

A review of Equations (16) and (24) shows that variable *e_Q_*_11_ closely matches relative reactive power error *e_Q_*_1_. When the adaptive virtual impedance is increased, |*e_Q_*_11_| becomes slightly smaller than |*e_Q_*_1_|.

Assuming that the reactive power droop coefficients of the two DG units are equal (*G_Q_*_1_ = *G_Q_*_2_) and the adaptive virtual impedance coefficients are the same (*K_Q_*_1_ = *K_Q_*_2_), then the equivalent line impedance of DG1 is greater than that of DG2. According to the analysis in Section 2.2, this implies *X*_1_ > *X*_2_, leading to the following relationships: *Q*_1_ > *Q*_2_ for reactive power, and *U*_1_ > *U*_2_ for output voltage magnitude. From Equation (24), we can infer that *e_Q_*_12_ > 0, *e_Q_*_11_ < 0, and therefore |e^*^_Q__1_| *<* |*e_Q_*_1_|. These outcomes verify that the proposed control scheme markedly reduces errors in reactive power allocation.

**Figure 9:**
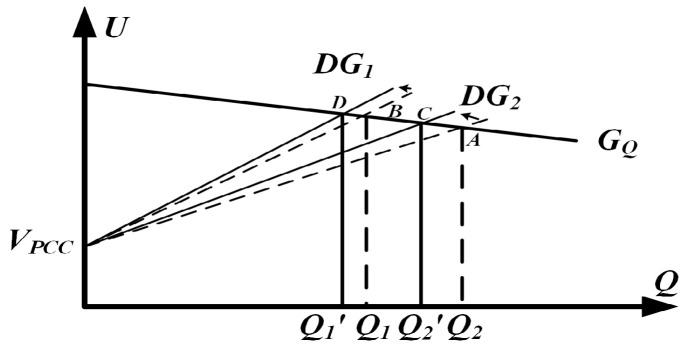
Reactive power sharing for the two DG units twice—dotted for operation without adaptive virtual impedance and solid for operation with adaptive virtual impedance.

Figure 9 depicts the *Q*–*U* profile under adaptive virtual impedance regulation. Equation (10) shows that the gradient of each curve is governed by the combined impedance of its branches. Under ideal conditions, the two DG units initially converge to operating points A and B. Due to differences in line impedance, adaptive virtual impedance is introduced to compensate for these deviations, resulting in the curves ultimately stabilizing at points C and D. As described by Equation (24), the addition of adaptive virtual impedance is effectively equivalent to adjusting the slopes of the *Q*–*U* curves, bringing them closer together. As shown in Figure 9, adaptive virtual impedance clearly improves the distribution of reactive power.

Implementing the adaptive virtual impedance scheme yields the following expression for each DG’s aggregate virtual impedance:

(25) $$\begin{array}{ll} L_{\mathrm{vir}} & =L_{v}+\Delta L_{vir}\\ & =L_{\nu }+\frac{k_{Q}}{s^{2}}(\frac{Q}{u}-\frac{Q^{*}}{u^{*}}) \end{array}$$

where *L_v_* is the baseline virtual inductance that maintains the overall line impedance inductive, Δ*L_vir_* is the adaptive term that adjusts this impedance in real time, and *L_vir_* denotes the aggregate virtual impedance assigned to each DG unit.

**Figure 10:**
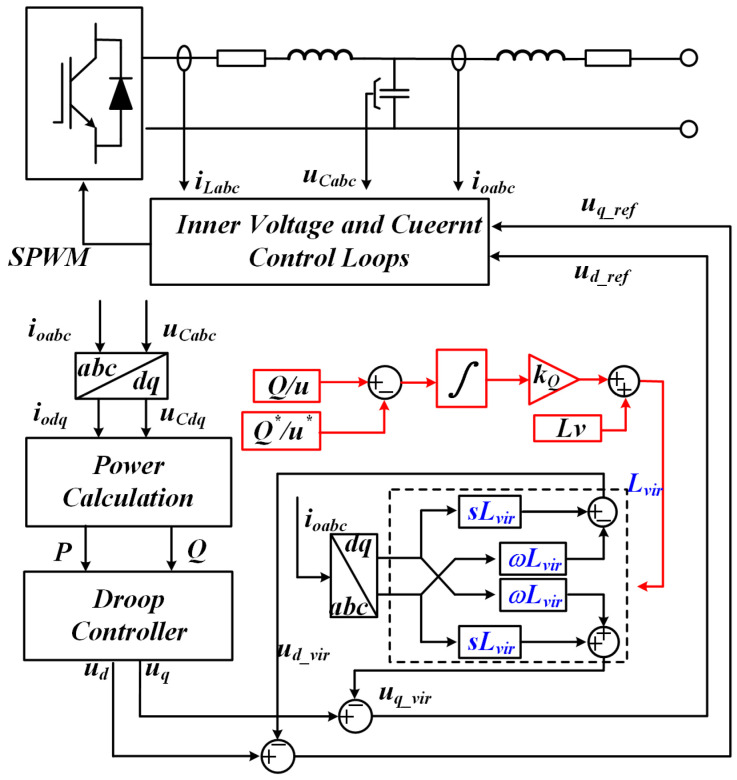
Block diagram illustrating the control architecture for virtual-impedance compensation.

Figure 10 illustrates the system control structure employing adaptive virtual impedance. The three-phase current and voltage output by the inverter are collected using current and voltage sensors. The PQ calculation is performed using Equations (4) and (5), and droop control is implemented in accordance with Equations (6) and (7). The adaptive virtual impedance value is applied to the dq axis to obtain the reference voltage, which is defined as follows: Ultimately, through the voltage-current dual closed-loop control, the three-level active neutral point clamped diodes are driven to conduct and turn off via SPWM [32].

(26) $$\left[\begin{array}{c} u_{d_{-}\mathrm{ref}}\\ u_{q_{-}\mathrm{ref}} \end{array}\right]=\left[\begin{array}{c} u_{d}\\ u_{q} \end{array}\right]-\left[\begin{array}{cc} sL_{\mathrm{vir}} & -\omega L_{\mathrm{vir}}\\ \omega L_{\mathrm{vir}} & sL_{\mathrm{vir}} \end{array}\right]\left[\begin{array}{c} i_{d}\\ i_{q} \end{array}\right]$$

## 4. Simulation and Experimental Analysis

To verify the feasibility of the proposed AVIDC strategy, a simulation model of an islanded microgrid comprising two DG units was developed. In this system, two inverter units share a common load, with each connected through feeder lines of unequal impedances. The main parameters and their corresponding values for the target system are presented in Table 1.

### 4.1. Simulation Analysis

**Figure 11:**
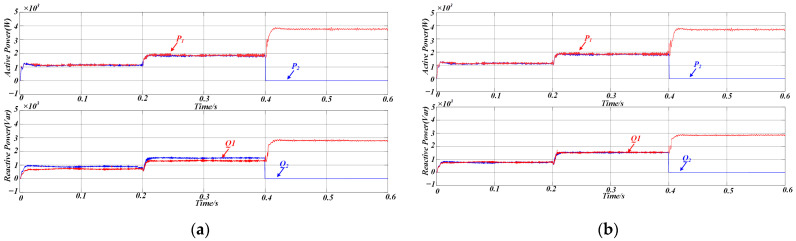
(**a**) Simulation results of power sharing using traditional droop control (**b**) Simulated power-sharing performance under the AVIDC strategy.

As shown in Figure 11, the power distribution under conventional droop control is illustrated for two DG units with identical rated powers. During the first stage (0 < t < 0.2 s), the microgrid is initially connected to Load 1 only. In the second stage (0.2 < t < 0.4 s), a sudden load change occurs as Load 2 is added to the system. In the third stage (0.4 < t < 0.6 s), DG2 is abruptly disconnected.

Under steady-state conditions, as depicted in Figure 11a, conventional droop control ensures acceptable active power sharing, even in the presence of a line impedance mismatch. However, there is a significant error in reactive power sharing, indicating that discrepancies in line impedance negatively impact the accuracy of power distribution.

Figure 11b summarizes the simulation results for the proposed control strategy when both DG units are rated at the same power level, and the test sequence replicates the events depicted in Figure 11a. This figure clearly shows that active and reactive power outputs are in a 1:1 ratio when the DG units have the same rated power, and that the output power maintains excellent consistency even when the load changes abruptly or the parallel inverter structure is altered. Corresponding error metrics for the conventional approach and the AVIDC scheme are listed in Table 2.

**Figure 12:**
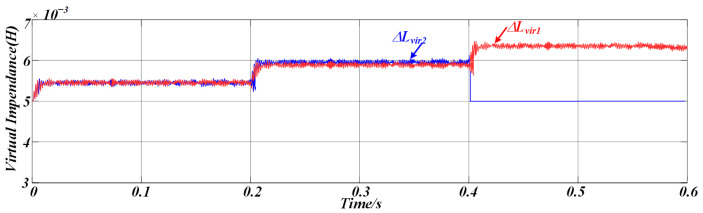
Simulation results of adaptive virtual impedance variation.

**Figure 13:**
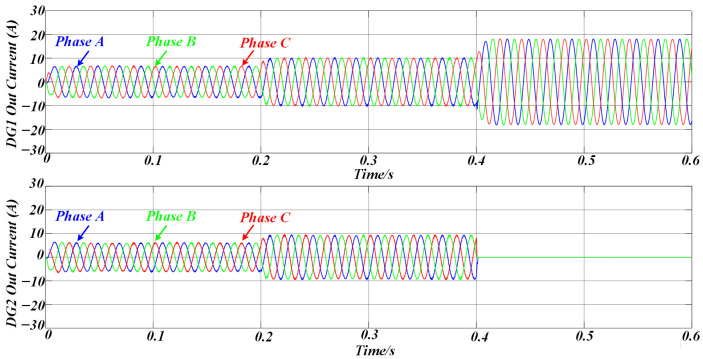
The simulation results of the three-phase output currents of DG1 and DG2 under AVIDC control.

Figure 12 illustrates the variation in the proposed adaptive virtual impedance at different stages to accommodate line impedance mismatches. Figure 13 shows the three-phase output current waveforms of the two DG units, which maintain excellent consistency in terms of amplitude and phase under the same rated power conditions, ensuring that they have the same active and reactive power output. The proposed strategy enables a rapid response to sudden load changes and variations in DG unit availability, ensuring that the output currents of both DG units remain aligned. This effectively suppresses circulating currents between the units.

Therefore, the simulation results confirm that the same value of reactive power can be output with the same rated capacity of the inverters, and the equalization of reactive power is achieved, thus proving the validity of the proposed method.

### 4.2. Experiments Demonstrate

**Figure 14:**
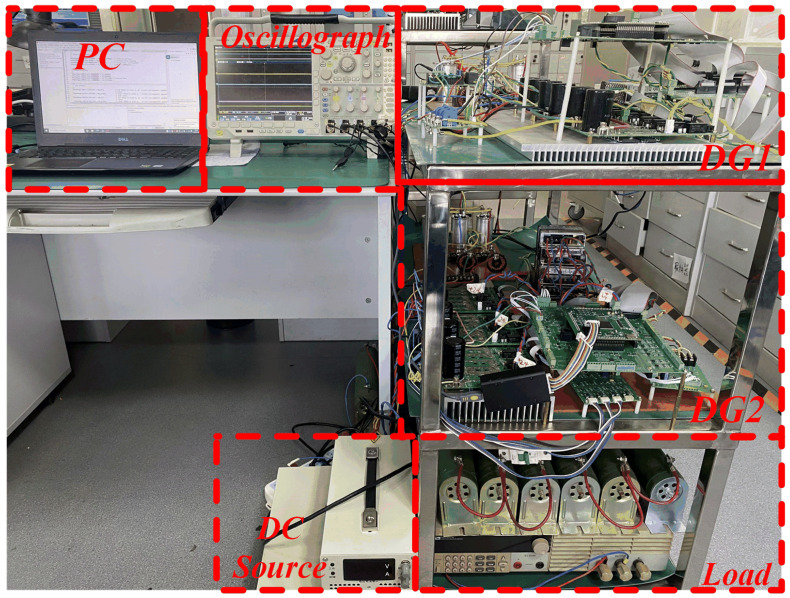
Experimental platform.

To verify the feasibility of the proposed strategy, a 5 kW three-phase DG system is designed and built. The prototype is shown in Figure 14, and its specifications are listed in Table 1. The DSP TMS320F28379D is employed as the main controller. To support higher voltages and power levels, STGW30V60DF power switches and an active neutral point-clamped three-level converter topology are employed.

**Figure 15:**
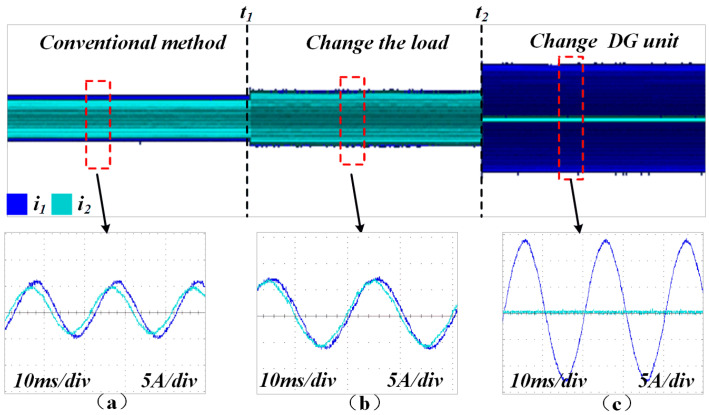
Output current waveforms of the two DG units under conventional control. (**a**) Conventional droop method (load1 is connnected). (**b**) Conventional droop method with changeable load (load1 and load2 are connected). (**c**) Conventional droop method with changeable DG units (DG1 is connected).

Figure 15 illustrates the current-sharing performance of the two DG units. When the conventional method is applied, inconsistencies in line impedance between the inverters result in discrepancies in both the amplitude and phase of the currents.

**Figure 16:**
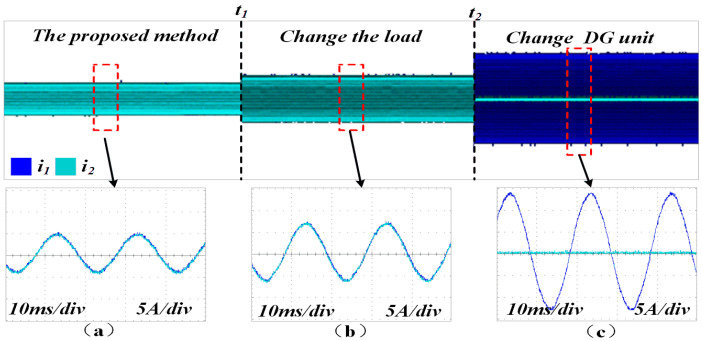
Output current waveforms of the two DG units under AVIDC control. (**a**) Proposed method (load1 is connnected). (**b**) Proposed method with changeable load (load1 and load2 are connected). (**c**) Proposed method with changeable DG units (DG1 is connected).

Figure 16 shows that by employing the proposed control method, the two DG units maintain strong consistency in both current amplitude and phase. Additionally, the method demonstrates excellent performance in response to sudden load changes at time *t*_1_ and structural changes in the microgrid at time *t*_2_.

**Figure 17:**
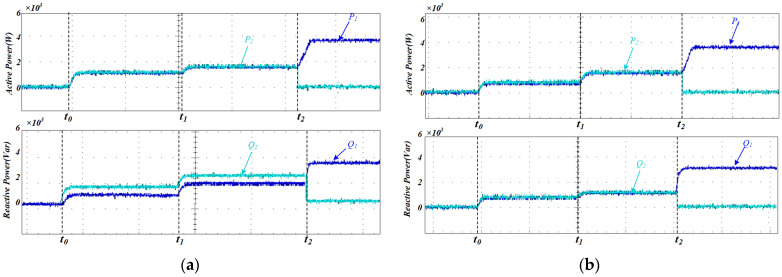
(**a**) Experimental evaluation of power-sharing efficacy under conventional droop control; (**b**) experimental investigation of power-sharing performance under AVIDC.

The active and reactive power outputs of each inverter under the conventional droop control strategy are shown in Figure 17a. It can be seen that while the active power of the parallel inverters is equally shared, disparities in line impedance among the inverters lead to deviations in reactive power distribution, preventing equal sharing of reactive power. Figure 17b illustrates the power-sharing performance of the two inverters after the proposed method is applied. The reactive power remains equally shared at t0 when both inverters supply the same load, at t1 when the load undergoes a sudden change, and at t2 when DG2 is disconnected, demonstrating the effectiveness of the proposed method.

**Figure 18:**
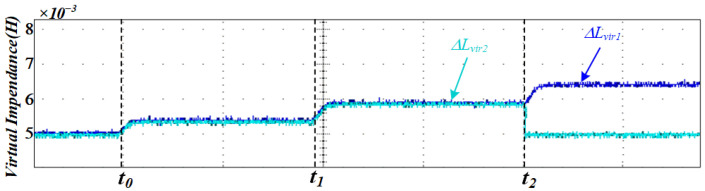
Experimental results of adaptive virtual impedance variation.

Figure 18 illustrates the adaptive variation of the virtual impedance under the proposed control method when the DG units have identical rated powers, enabling effective averaging in reactive power sharing.

From the experimental analysis, it can be concluded that using the proposed control method, the value of virtual impedance can be adjusted in real time with changes in the working conditions, which offsets the difference in line impedance. The amplitude and phase of the output current of the inverter are maintained consistently, which ensures the consistency of the output power.

Table 3 compares the proposed strategy with those of previous studies. Compared with previous methods, the proposed method exhibits better reactive power allocation performance with less reactive power sharing error in the face of sudden load changes and inverter structure changes. Moreover, it relies solely on the local measurement data of the inverters and exhibits excellent scalability.

## 5. Conclusions

In islanded microgrids, mismatches in feeder impedance often lead to an uneven sharing of reactive power among parallel inverters and trigger circulating currents. To counteract these drawbacks, we first quantified the resulting reactive power error and then proposed an AVIDC strategy. The scheme dispenses with any communication links and relies exclusively on measurements local to each inverter. By continuously integrating the deviation between the instantaneous reactive power to voltage ratio and its rated power to voltage reference, the controller synthesizes a time-varying virtual impedance, thereby retuning the inverter output in real time and restoring balanced reactive power sharing across the microgrid. The results show that compared with the conventional droop control approach, the proposed strategy significantly improves the accuracy of reactive power sharing and maintains strong regulation performance even under sudden load changes and microgrid structural variations. This control method is of great significance in ensuring accurate power sharing in islanded microgrids with mismatched line impedances among distributed generation units. Finally, both simulation and experimental results verify the feasibility of the proposed control strategy.

## Figures and Tables

**Table 1:** System Parameter.

System Parameter	Values
Microcontroller	DSPTMS320F28379D
IGBT Module	STGW30V60DF
Rated line to line RMS voltage level, *U*_0_	155 V
Nominal frequency, *f*_0_	50 Hz
LC filter, *R*_f_, *L*_f_, *C*_f_	0.1 Ω, 3 mH, 30 μF
DC link voltage, *U*_dc_	400 V
Low-pass filter, *ω*_r_	50 rad/s
Switching frequency, *f*_s_	10 kHz
Line 1 impedance, *R*_1_, *L*_1_	0.05 Ω, 0.05 mH
Line 2 impedance, *R*_2_, *L*_2_	0.1 Ω, 0.1 mH
*G*_P_	5 × 10^−6^
*G*_Q_	4 × 10^−5^
*K*_pv_, *K*_iv_, *G*_i_	0.02, 30, 20
*L*_v_	5 mH
*k*_Q_	0.00027
Load1 *R*_1_, *L*_1_	10 Ω, 10 mH
Load2 *R*_2_, *L*_2_	20 Ω, 15 mH

**Table 2:** Simulation-based comparison of reactive power-sharing deviations.

System Operating Mode	Error/%	DG Parallel Operation	Change the Load	Change DG Unit
Traditional control methods	Qerr_1	40.5%	38.9%	22.4%
	Qerr_2	30.8%	27.6%	-
The proposed method	Qerr_1	8.5%	2.1%	1.1%
	Qerr_2	6.9%	2.6%	-

**Table 3:** Reactive power sharing deviations: AVIDC versus alternative control schemes.

System Operating Mode	Communication Mode	Scalability	Error/%	DG Parallel Operation	Change The Load	Change DG Unit
Distributed adaptive virtual impedance control [25]	CAN Communication	Yes	Qerr_1	17.4%	13.4%	9.6%
			Qerr_2	16.5%	11.7%	-
Weight particle swarm optimization algorithm [33]	No	No	Qerr_1	13.5%	8.4%	5.9%
			Qerr_2	11.4%	6.8%	-
Equivalent input disturbance [7]	No	No	Qerr_1	18.6%	15.4%	12.1%
			Qerr_2	17.7%	13.5%	-
AVIDC	No	Yes	Qerr_1	9.1%	2.3%	1.4%
			Qerr_2	7.6%	3.1%	-

## Data Availability

The data presented in this study are available in this article.
